# Regenerative Stem Cell Therapy for Neurodegenerative Diseases: An Overview

**DOI:** 10.3390/ijms22042153

**Published:** 2021-02-22

**Authors:** Farzane Sivandzade, Luca Cucullo

**Affiliations:** 1 Department of Biological Sciences, Oakland University, Rochester, MI 48309, USA; fsivandzade@oakland.edu; 2 Department of Foundation Medical Studies, Oakland University William Beaumont School of Medicine, Rochester, MI 48309, USA

**Keywords:** stem cells, therapy, regenerative, neurodegenerative diseases, Parkinson’s disease, Huntington’s disease, Alzheimer’s disease, amyotrophic lateral sclerosis

## Abstract

Neurodegenerative diseases resulting from the progressive loss of structure and/or function of neurons contribute to different paralysis degrees and loss of cognition and sensation. The lack of successful curative therapies for neurodegenerative disorders leads to a considerable burden on society and a high economic impact. Over the past 20 years, regenerative cell therapy, also known as stem cell therapy, has provided an excellent opportunity to investigate potentially powerful innovative strategies for treating neurodegenerative diseases. This is due to stem cells’ capability to repair injured neuronal tissue by replacing the damaged or lost cells with differentiated cells, providing a conducive environment that is in favor of regeneration, or protecting the existing healthy neurons and glial cells from further damage. Thus, in this review, the various types of stem cells, the current knowledge of stem-cell-based therapies in neurodegenerative diseases, and the recent advances in this field are summarized. Indeed, a better understanding and further studies of stem cell technologies cause progress into realistic and efficacious treatments of neurodegenerative disorders.

## 1. Introduction

Neurodegenerative disorders, such as Parkinson’s disease (PD), Alzheimer’s disease (AD), Huntington’s disease (HD), amyotrophic lateral sclerosis (ALS), and frontotemporal dementia (FTD), are characterized by a progressive loss of structure, function, or number of neurons in the brain or spinal cord. Unfortunately, the currently available treatment options are insufficient in arresting the neurodegenerative processes [1]. The complexity of the mechanisms associated with neuronal loss and the contradicting physiological causes of these diseases significantly hinder our understanding of the pathogenic processes and the consequential development of effective treatments [2]. Moreover, difficulty in targeting the widespread neuronal cell death, coupled with the lack of robust regenerative capacity of the central nervous system (CNS) and the enormous limitations for the vast majority of drugs (98% of small-molecule drugs and 100% of large-molecule drugs) regarding crossing the blood–brain barrier (BBB) further adds to the difficulty of treating these diseases [3,4,5,6,7]. The loss of quality of life, the cost of care, and the lack of effective therapies are an enormous burden for over 7 million people in the USA living with these neurodegenerative diseases [8]. 

Stem cell therapy, also known as regenerative therapy, improves the repair response of dysfunctional and damaged tissue using stem cells or their derivatives. The objectives of stem cell therapies typically focus either on cellular replacement or on providing environmental enrichment. Stem cell therapy has revolutionized medicine over the years since its therapeutic applications have provided invaluable and attractive options for treating numerous disorders, including neurodegenerative diseases [9]. The potential of stem cell therapy in neurodegenerative diseases was first examined in the 1980s when patients suffering from PD were treated with fetal mesencephalic tissue transplantation [10]. Nowadays, stem cell therapy offers promising strategies for treating almost all forms of neurodegenerative disorders. These strategies involve the regeneration of neural tissue, stabilizing the neuronal networks, providing neurotrophic support, and alleviating neurodegeneration at different neuronal circuitry levels [9]. Scientists are continually trying to find sturdy, safe, and readily available stem cell sources while refining and/or developing new delivery methods to improve the treatment’s efficiency and effectiveness and reduce the side effects [11]. 

This review provides encompassing information on the various types of stem cells and then discusses the existing data, progress, and status of using stem cells to treat neurodegenerative diseases. We also describe the remaining challenges associated with transitioning stem cell therapies from bench to bedside. 

## 2. Stem Cell Classifications

**Figure 1:**
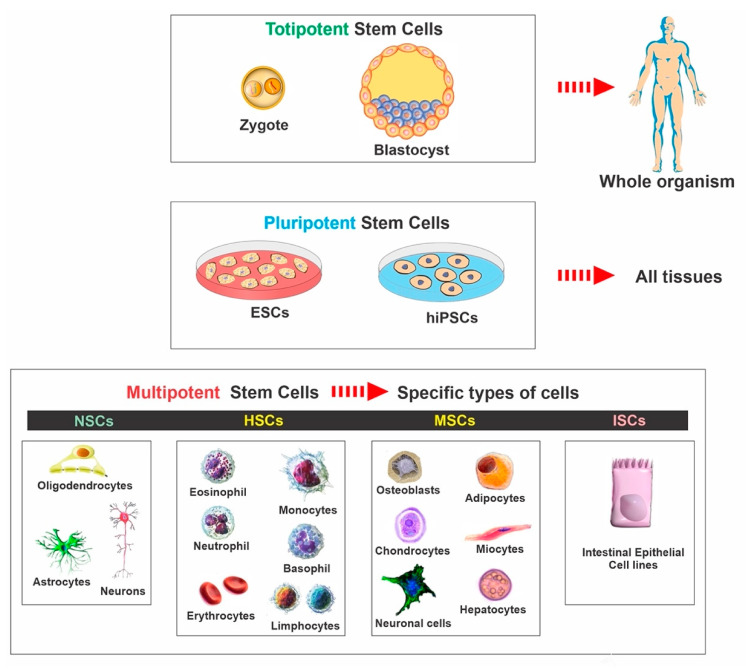
Classification of stem cells: ESCs—embryonic stem cells, hiPSCs—human induced pluripotent stem cells, NSCs—neural stem cells, HSCs—hematopoietic stem cells, MSCs—mesenchymal stem cells, ISCs—intestinal stem cells.

Stem cells are characterized by the capacity to proliferate, self-renew, and differentiate into various mature cell lineages. There are different classifications of stem cells, including embryonic stem cells (ESCs), induced pluripotent stem cells (iPSCs), mesenchymal stem cells (MSCs), and neural stem cells (NSCs). The classification is based on the range of possible cell type production and derivation methods. Therefore, it is essential to understand the characteristics of the various available stem cell types and the potential effect of cellular therapies on disease mechanisms (see Figure 1). The rationale for utilizing each type of stem cell depends on the desired applications and outcomes since each type possesses individual qualities and advantages. In the following paragraphs, we summarized the various types and general aspects of stem cells used in basic research and clinical trials (see Table 1).

### 2.1. Embryonic Stem Cells 

**Figure 2:**
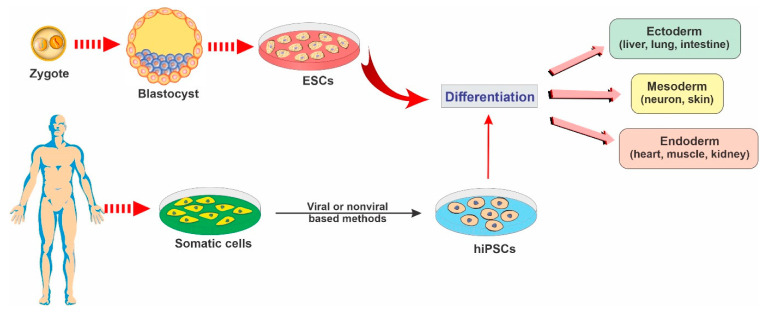
Representative diagram depicting the main types of stem cells and their potential to differentiate into various lineages.

ESCs are a class of pluripotent stem cells derived from the inner cell mass of blastocysts (an embryo that has been left to develop for 5 to 6 days and presents a relatively complex cellular structure formed of approximately 100–200 cells; see Figure 2). ESCs offer promising avenues for research due to their ability to self-renew indefinitely and differentiate into almost all cell types of the central nervous system. These cells are currently being used as an invaluable cell source of human neuronal progenitors in large quantities and high purity in various research areas related to neurodegenerative diseases [12]. 

Researchers are currently focusing heavily on the therapeutic potential of ESCs. Although ESCs offer new means of treatment, it still raises some thorny ethical and religious restrictions since it involves destroying human embryos [4,13]. Additionally, there are several medical concerns associated with all novel ESC therapies in translational medicine, such as the significant risk of immunorejection in the host patient, as well as tumor formation and cancer as a result of the persistence of nondifferentiated cells undergoing malignant transformation and genetic instability following a prolonged time in culture [14].

### 2.2. Induced Pluripotent Stem Cells 

iPSCs are a type of pluripotent stem cells that are artificially derived from non-pluripotent, adult somatic cells, including fibroblasts, hepatocytes, circulating T cells, and keratinocytes, by forcing the expression of genes and transcription factors that maintain embryonic stem cells [15]. These reprogrammed cells now provide a promising strategy for producing unlimited autologous neurons for transplantation in neurodegenerative patients [2]. iPSCs can be converted into mature functional neural lineages using an optimized differentiation method, which widens the scope of its potential applications in the studies of the mechanisms underlying various neurodegenerative disorders and the screening of novel therapeutic targets [16,17]. For example, in a patient with a neurodegenerative disease, a pluripotent cell can be taken from the skin or blood (see Figure 2). The resulting iPSCs can become a reliable source for generating those neural cells affected by degenerative brain disease [18]. 

One of the main distinct advantages of iPSCs is the lack of ethical and religious implications because cells can be produced without oocytes or embryos. Another significant merit of iPSCs is that they can be generated from the patients themselves, thus providing a valuable avenue for autologous cell transplantation with no risk of immune rejection and no need for immunosuppressive agents [19,20]. iPSCs offer potential clinical advantages due to more straightforward harvesting methods and fewer possible side effects, with better specific terminally differentiated cell phenotypes. However, the differentiation of IPSCs into mature neurons is more complicated than for ESCs. Like ESCs, there is still the risk of tumor formation due to unwanted viral integration, causing chromosomal disruptions and mutations and low reprogramming efficiency during these cells’ production [14,21]. Hence, the clinical application of IPSCs in neurodegenerative diseases is still not feasible yet, owing to the lack of in-depth research evaluating its therapeutic safety among human subjects.

### 2.3. Mesenchymal Stem Cells 

MSCs, which are traditionally found in the bone marrow, umbilical cord, adipose tissue, and spleen, are adult, self-renewing, multipotent stem cells that can differentiate into various cell types, including bone, cartilage, fat, and muscle [22]. MSCs have enormous therapeutic potential and could be an ideal source for cell transplantation in neurodegenerative diseases due to their excellent self-renewal capacity while maintaining multipotency [23]. MSC-derived functional neurons appear to be more promising regarding neurodegenerative diseases than ESCs due to the relatively easy collecting methods and fewer related ethical, religious, and immunorejection concerns [24]. Furthermore, MSCs do not organize tumors like other primitive stem cells, such as ESCs [25]. Thus, the promising abilities of MSCs present them as an attractive platform for research into neurodegenerative disorders. Several studies have also indicated that MSCs might possess the ability to cross the BBB, which is crucial for the proper delivery of neurotherapeutic agents into the CNS [26,27]. It has been shown that MSCs can cross the BBB through paracellular pathways, despite the presence of tight junctions that would normally block such passages [28]. There are preclinical studies and ongoing clinical trials currently assessing the therapeutic effectiveness of MSCs in various neurodegenerative diseases. MSCs are delivered via either intracerebral or intrathecal injections. Following transplantation, MSCs initiate their neuroregenerative function, including promoting neuronal growth, producing neurotrophic factors, stimulating endogenous neurogenesis, activating microglia, suppressing inflammation, and decreasing apoptosis and free radicals [18]. MSCs can also secrete angiopoietin-1, angiogenic cytokines, and extracellular matrix components, thus improving angiogenesis and promoting the recruitment of neural progenitor cells (NPCs) [29].

### 2.4. Neural Stem Cells 

NSCs are multipotent stem cells in brain tissue that are more specialized than ESCs. NSCs have a decreased potential for self-renewal and usually differentiate into only limited cell lineage of the brain tissue, including oligodendrocytes, neurons, and astrocytes [13,30] (see Figure 2). NSCs can be derived from various regions of both the embryonic and the human fetal brain or the brain tissue of patients undergoing surgical therapies [30,31,32,33]. 

The transplantation of NSCs to other brain regions is considered a possible therapeutic avenue for the treatment of many neurodegenerative diseases [1,34]. For example, NSCs can play a role in gliogenesis by releasing bioactive molecules that regulate neuronal excitability, synaptic activity, and plasticity [35]. NSCs can also generate and release synergistic and antagonistic molecules, triggering intracellular NSC regulatory mechanisms, such as transcription factors, epigenetic responses, and metabolism [36]. Furthermore, NSCs can establish synaptic connections with surrounding neurons, integrate into existing circuitry, and repair the impaired network [37]. Of note, unlike ESCs, NSCs are considered genetically stable and less tumorigenic. The low self-renewal potential of NSCs can be resolved via the genetic modification of these cells to produce immortalized NSCs with enhanced proliferative potential [38]. However, there are still significant obstacles for the therapeutic application of NSCs due to the inevitable possibility of immunological incompatibility in allogeneic transplantation, limited sources, difficulties in isolating these cells, limited proliferation and expansion, and ethical and religious issues [39].

## 3. Neurodegenerative Diseases and Stem Cell Therapy Strategies for Regeneration 

Neurodegenerative diseases, including PD, AD, HD, ALS, and FTD, are disorders of protein homeostasis characterized by the loss of specific neuronal populations and inclusion bodies consisting of insoluble and unfolded proteins. This pathogenic process leads to the progressive loss of sensation, cognition, motor neurons, and gradual paralysis. Despite billions of dollars in clinical trials and tremendous progress in understanding the mechanism of neurodegenerative disorders, there are still no detectable biomarkers or effective drugs to slow these diseases’ progress.

Although stem cell therapy is still in its infancy, it has become a safe, engaging, and beneficial strategy to be tested in treating neurodegenerative diseases [40]. The first objective of stem cell therapy for neurodegenerative disorders includes deriving specific neuronal subtypes and recapitulating a neural network similar to the one lost in the disease. Another approach for treating neurodegenerative disorders is creating environmental enrichment to support host neurons by producing neurotrophic and scavenging toxic factors or building auxiliary neural networks around affected areas [8]. Many strategies utilize stem cells to provide de novo synthesis and the delivery of neuroprotective growth factors (such as glial-derived neurotrophic factor (GDNF), brain-derived neurotrophic factor (BDNF), insulin-like growth factor 1 (IGF-1), and vascular endothelial growth factor (VEGF)) at the site of disease for environmental enrichment.

**Figure 3:**
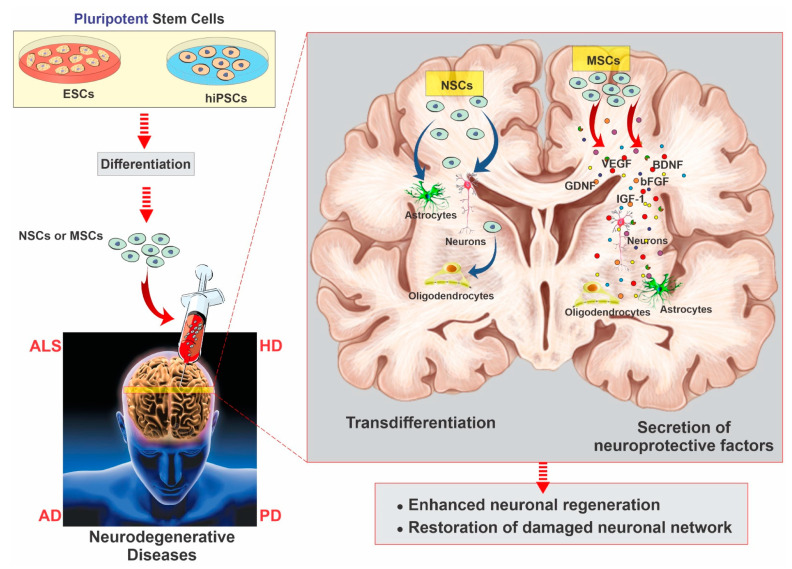
Neurodegenerative disease modeling of hiPSCs and ESCs. These cells can be differentiated into neuronal progenitor (NPCs) and MSCs, from which brain cells, such as oligodendrocytes, astrocytes, and different neuronal and glial lineages, can be generated. Note also that the trophic action of MSCs, including the secretion of growth and neurotrophic factors, can act as a coadjuvant to nervous tissue regeneration by promoting angiogenesis, neurogenesis, and immunomodulation. AD: Alzheimer’s disease, ALS: amyotrophic lateral sclerosis, HD: Huntington’s disease, PD: Parkinson’s disease; BDNF: brain-derived neurotrophic factor, bFGF: basic fibroblast growth factor, IGF-1: insulin-like growth factor 1, GDNF: glial-derived neurotrophic factor, VEGF: vascular endothelial growth factor.

In recent years, investigators have carried out extensive efforts to produce neurons and glial cells from various stem cells and exploit other beneficial stem cells’ aspects to treat neurodegenerative diseases. Multiple sources of stem cells have been examined to determine the most efficacious and productive method for the stem cell therapy of neurodegenerative diseases [41,42,43]. Most of the research on stem-cell-based therapy for neurodegenerative diseases has been conducted preclinically in animal models. These studies have shown that stem cells could impact endogenous cells, promote the functional recovery of nervous tissue, differentiate into neuronal and glial cells, and decrease motor impairments [44,45]. Many clinical studies are investigating different aspects of stem cell therapies for neurodegenerative disorders [44,46,47]. So far, the data seem to support the results obtained from preclinical studies to some extent. For example, there is a consensus of data showing that neuroprotection is achieved by the secretion of growth factors (such as brain-derived neurotrophic factor, glial cell line-derived neurotrophic factor, and nerve growth factor), which is the fundamental mechanism for the observed improvements in neurodegenerative disorders [48]. Additionally, there is significant evidence showing that stem cell therapies can enhance neurogenesis in neurodegenerative patients [49,50,51] (see Figure 3). 

Moreover, selecting the appropriate stem cell type and understanding the mechanism of support and the specific neuronal pathology are the main steps in developing and translating stem cell therapies from the bench to patients. For example, cellular replacement may be useful in PD, where a specific neuronal subpopulation is lost. In contrast, ALS is most likely to benefit from cellular therapies that enrich the local spinal cord environment to support the remaining motor neurons [8]. Hence, in the following paragraph, we discussed the mechanism of neurogenesis and the pathophysiology of common neurodegenerative diseases. Next, we describe the currently supported approaches and successful progress in translating stem cells from the bench to the bedside regarding curing those specific neurodegenerative diseases.

### 3.1. Stem Cell Therapy in Parkinson’s Disease

Dopamine (DA), a key neurotransmitter that transmits signals between neurons, plays an essential role in motor control. PD is the second-most-common age-related progressive neurodegenerative disorder caused by DA deficits in the striatum due to the destruction of DA-producing neurons located in the substantia nigra [52,53,54]. The initial symptoms are sometimes barely noticeable, such as tremors affecting one hand or the slowing of movement. As the disease progresses, controls over movement are entirely compromised, and patients may present symptoms such as muscle tremors, muscle rigidity, slowing of voluntary movement, postural instability, bradykinesia, and other motor dysfunctions, usually in the fifth to seventh decade of life [24]. To date, approximately 60,000 Americans are diagnosed with PD yearly, and more than ten million people worldwide are suffering from PD [55].

A specific diagnosis of PD includes the presence of Lewy bodies in the brains, which are abnormal protein aggregates developing inside nerve cells and found in both familial and sporadic PD patients. The main constituent of Lewy bodies is α-synuclein protein, a small protein with 140 amino acids that are abundantly found in presynaptic nerve terminals and play a role in synaptic transmission and DA level adjustments. α-Synuclein primarily affects tyrosine hydroxylase phosphorylation, activity, and DA transporter expression on the cell membrane [56].

The current treatment options for PD include deep brain stimulation or therapies to increase DA levels by providing a DA precursor (Levodopa) to compensate for the deficit in DA caused by the destroyed dopaminergic neurons [57]. Although current medications have proven helpful in alleviating the symptoms, they cannot reverse the significant loss of dopaminergic neurons over time as the disease progresses. On the other hand, several different factors, including protein folding and dysfunction of the ubiquitin-proteasome pathway, mitochondrial dysfunction, and oxidative stress, have been discovered to contribute to the onset and progression of the disease [2,58]. Thus, the heterogeneity in pathology and underlying causes of PD makes its treatment challenging.

Over the past two decades, researchers have been looking for alternative strategies to supplement DA by replacing the dopaminergic neurons lost in the disease with stem-cell-derived equivalents. ESCs, NSCs, and iPSCs are among the stem cell typologies that scientists are working with to induce their differentiation into the mature dopaminergic cell [59]. Stem cell therapy for the treatment of PD has demonstrated some success in animal models. Clinical trials have just started employing the transplantation of brain cells isolated from human fetuses into PD-diagnosed patients to assess the procedure’s efficacy while minimizing the possible side effects. For example, Schwarz et al. used human-fetus-derived dopaminergic neurons and transplanted them into PD patients’ depleted striata [60].

Furthermore, Takagi et al. demonstrated functional recovery after successfully transplanting dopaminergic neurons from monkey ESCs into PD patients’ brains [61]. Another study also confirmed the influential role of undifferentiated ESCs in functional recovery by differentiating dopaminergic neurons in a PD rat model [55]. Two other clinical studies using human ESCs are ongoing in Australia and China, where their preclinical studies’ results were reported [62,63]. Specifically, Garitaonandia et al. [62] described the preclinical tumorigenicity and biodistribution safety in vitro before conducting a phase I clinical trial to evaluate the safety and tolerability of parthenogenetic stem cells for the treatment of PD [62]. In the other study, Wang et al. [63] investigated the ability of human ESCs regarding neuronal differentiation and their potential to produce DA neurons. Using nonhuman primate models of Parkinson’s disease, the investigators tested these DA neurons’ safety and efficacy to assess the optimal processes for employing stem cell therapy in PD patients [63].

Moreover, ESCs have shown excellent outcomes in mice models due to their ability to form dopaminergic neurons for treating PD, which has not been accomplished while using adult neural stem cells. Moreover, in rat models with spinal cord injuries, ESCs showed migration into the parenchyma and spinal cord. They led to partial motor recovery, giving rise to the ultimate goal of reversing motor degeneration in PD. While these studies initially revealed promising results, they were banned due to ethical and religious concerns and the risk of tumorigenesis [64]. Besides ESCs, NSCs have also shown promising results. Because NSCs have a dopaminergic interneuron phenotype, they can release DA, thus alleviating PD symptoms. Deleidi et al. reported the successful differentiation of adult NSCs into functional midbrain dopaminergic neurons in the subventricular zone (SVZ) to improve motor deficits in a PD rat model [65]. Yasuhara et al. transplanted human NSCs into a rat model of PD, thus alleviating the disease’s symptoms [66]. In another model, nuclear-1-receptor-engineered NSCs derived from the SVZ and differentiated into dopaminergic neurons in the rat model of PD resulted in reversed behavioral deficits in those animals. 

MSCs have also been shown to reduce dopamine depletion and rebuild the damaged striatal dopaminergic nerve terminal network in a PD animal model [18]. A more recent in vivo study revealed that stem cell therapy with human amniotic fluid stem cells and MSCs ameliorated bladder dysfunction [67]. In a pilot clinical trial on seven patients who received MSC transplantations in the lateral ventricles’ walls, Venkataraman et al. reported promising functional recovery with no adverse effects and improved dyskinesias [68]. Moreover, in a recent study on 53 patients diagnosed with PD, engineered MSCs differentiated into dopaminergic cells were directly implanted into an artery that feeds the substantia nigra [55]. The results revealed that intra-arterial autologous stem cell implantation is a safe and beneficial procedure that eliminates the risks of tumor formation and immunological attacks.

iPSCs have proven to be very useful for promoting dopamine replacement in patients with PD. Several studies have shown that dopaminergic neurons derived from iPSCs grafted into PD model systems can survive and integrate into the host network with remarkable functional improvements [69]. Other studies also demonstrated that the transplantation of reprogrammed iPSCs into dopaminergic neurons improved functional deficits and cell integration in vivo [70]. These data suggest that iPSCs provide a suitable approach for autologous cell-based therapy in PD [71]. Through a preclinical study, Doi et al. confirmed the safety and efficacy of iPSC-derived DA progenitors for providing long-time survival and functionality of the grafted cells as DA neurons for clinical trials on PD patients [72]. iPSCs offer many advantages, including minimal immune response from the host post-transplantation and a noninvasive method of iPSC isolation from peripheral blood cells [73,74]. However, a drawback of iPSCs is that these cells need to be induced to become dopamine-producing neurons, where the process can be quite laborious and expensive [75].

Although cellular replacement is considered a viable approach for treating PD, environmental enrichment through growth factor delivery may also support existing DA neurons’ ability to slow or prevent further degeneration. More recent work has focused on encapsulating the desired cell line into a semipermeable polymer, along with a pool of neurotransmitters and neurotrophic factors. This approach combines cellular replacement and environmental enrichment strategies. For example, the transplantation of NSCs derived from the SVZ and the nerve growth factor (NGF) in the striatum of animal PD models showed remarkable recovery [46]. Taken together, the combination of cellular replacement and environmental enrichment may improve the efficacy of stem cell therapies for PD treatment. Overall, PD has emerged as a well-suited neurodegenerative disease for stem cell therapy, although careful considerations need to be made when evaluating the potential risks of graft-induced dyskinesias [40,75]. Politis et al. indicated an increased serotonin-to-DA-transporter ratio in the grafted striatum after 14 years of transplantation [76].

### 3.2. Stem Cell Therapy in Alzheimer’s Disease

AD is the most common progressive neurodegenerative disorder characterized by the degeneration of synapses and loss of neurons in the hippocampus and the neocortex [52]. AD progression leads to memory decline, judgment impairment, disorientation, loss of language and problem-solving skills, and in the advanced stage, dementia and eventually death [77,78]. AD occurs in two forms: (1) early-onset familial (due to genetic mutations), affecting patients less than 65 years old, and (2) late-onset sporadic forms involving patients over 65 years of age. The late form of AD seems to be associated with genetic polymorphisms, aging, type 2 diabetes, traumatic brain injury, stroke, and Down syndrome [55]. 

While AD’s exact pathology remains unclear, the pathologic hallmarks of AD, which lead to decreased synaptic signaling and eventually neuronal death, involve the formation of extracellular senile plaques (SPs, composed of small Aβ peptides) and intracellular neurofibrillary tangles (NFTs). NFTs deposited inside the cell are made of hyperphosphorylated tau proteins that form tangles after dissociating from destabilized microtubules [24]. Aβ plaques, which are deposited outside of neurons, are formed from the amyloid precursor protein fragments. In addition to massive neuronal death and synaptic dysfunction resulting in a profound shrinkage in brain volume and weight, especially in the telencephalon, imbalanced mitochondrial activity is another pathogenesis of AD [79]. Imbalanced mitochondrial activity leads to decreased mitochondrial adenosine triphosphate concentrations, oxidative stress, and increased intracellular calcium levels [56].

Alzheimer’s is the sixth-leading cause of death, with a substantial economic burden on society [80,81]. Approximately 5.3 million Americans suffer from AD, including 5.1 million individuals over the age of 65 [82]. Current therapeutic strategies have focused on the breakdown of Aβ plaques and NFTs and the reduction of reactive oxygen species (ROS) in the brain [24]. AD’s current treatment option focuses on promoting cell survival, substituting the lost neurons, and regulating neurotransmitter activity. However, none of the medications currently approved by the U.S. Food and Drug Administration (FDA) are curative and fully efficient across patients [78].

Evidence supports that stem cell therapies may play a beneficial role in slowing AD’s progression through enhancing neurogenesis, replacing lost neurons, and providing environmental enrichment. Human NSCs have been proven to be a promising source, improving synaptic plasticity, reducing the pathology’s burden, and ameliorating spatial learning and memory dysfunction by increasing the expression of multiple cognition-related proteins in vivo in AD mouse models [83,84,85]. Of note, no reduction in Aβ or tau pathology was diagnosed, indicating that the regenerative capacity of NSCs could help to balance the degenerative processes occurring in the AD brain but does not treat the underlying pathology [86]. NSCs transplanted in the hippocampus improved memory deficits in an AD mouse model by releasing BDNF and improving cognitive function [9]. Furthermore, transplanted NSCs migrate and differentiate into various brain cells (including cholinergic neurons, astrocytes, and oligodendrocytes), stimulate endogenous neural precursors, promote structural neuroplasticity, inhibit pro-inflammatory cytokines, suppress neuronal apoptosis, and release growth factors. Kern et al. also revealed that NSCs transplanted into the hippocampus of aged Down syndrome mice promote a striking decrease in the tau/reelin-positive granule density [87]. Overall, the mechanism of action underlying how NSCs promote neurogenesis and cognitive function is still unclear. Furthermore, the generation of non-neuronal glial cell types with NSC transplantation is still a limiting factor in AD treatment with stem cell therapies [88].

The use of patient-specific iPSCs in the treatment of AD is still a work in progress. Scientists have employed a novel way to directly create functional neurons from AD patients’ skin cells converted into ESCs [89,90]. In a recent study, iPSC-derived cholinergic neuronal precursors transplanted in the hippocampus of transgenic AD mice were differentiated into mature cholinergic neurons and reversed spatial memory impairment. However, the use of iPSCs for neuroreplacement in AD treatment may carry a non-negligible risk of tumor formation since these cells have a highly proliferative phenotype [88]. Fortunately, adult cells can be programmed into mature neurons without reversing them into their stem cell phenotypic form, thus eliminating the tumor formation risk [91,92]. 

Several studies reported that MSCs stimulate neurogenesis, synaptogenesis, neuronal differentiation and influence amyloidogenesis and/or microglial activation, thus reducing Aβ accumulation and cognitive recovery [93,94,95,96,97]. For example, Kim et al. and Ma et al. indicated that MSCs could alleviate memory impairments and reduce the amount of Aβ in AD mouse models by upregulating interleukin-10 and vascular endothelial growth levels factor while modulating microglial activation in the brain [98]. Moreover, Kan et al. showed that MSCs transplanted into an AD model induced the proliferation, differentiation, and maturation of endogenous NSCs toward a neuronal phenotype [99]. Martinez-Morales et al. also reported that MSCs could produce neurotrophic factors to stimulate endogenous neurogenesis, angiogenesis, and neuronal defense systems [100]. The clinical trials for AD treatment are still in their infancy, where the FDA approved the first clinical trial of MSCs for AD treatment in 2015. Similar trials are currently underway or under development in Europe and Asia to assess the safety, tolerability, and preliminary efficacy of human MSCs in patients with mild-to-moderate AD [101].

Recently, combining stem cells with NGF was recognized as a useful strategy for preventing cell death, stimulating the growth of cholinergic neurons, and facilitating the generation of specific neural populations in AD treatment. NGF gene therapy has been studied in many animal models and has led to successful clinical trials in AD patients [102]. 

### 3.3. Stem Cell Therapy in Huntington’s Disease

HD is a fatal progressive neurodegenerative disorder of autosomal dominant inheritance characterized by the loss of GABAergic inhibitory spiny neurons in the striatum of the forebrain, accompanied by degeneration in the cortex, brain stem, and hippocampus [52]. The HD’s pathophysiology is focused on the abnormal expansion of cytosine-adenine-guanine (CAG) encoding repeats at the N-terminal of the huntingtin protein (Htt), leading to the preferential loss of medium spiny neurons of the striatum and giving rise to involuntary motor activity, dementia, and cognitive and emotional deficits [8]. The progress of HD typically occurs in the fourth to fifth decade of life, with a disease course of approximately 20–30 years. Despite the known genetic basis and disease mechanisms of HD, the identification of effective therapies remains elusive.

Stem-cell-based therapeutic approaches have received considerable attention as potential treatments for HD. The objective of stem cell therapy for HD is to replace the damaged or lost neurons and modify the mutant genes containing the expanded CAG repeats. Based on recent studies, NSCs have been the type of stem cells most used for HD treatments. NSCs have been derived and induced from various sources, such as the brain itself and the somatic cells of HD patients. Despite the early stage of stem-cell-based preclinical and clinical trials in HD, there is convincing evidence regarding stem cells’ transplantation or their derivatives in HD animal models. Initial stem cell therapies focused on ESC-derived NSCs grafted into HD models have demonstrated the integration of motor neurons and circuitry formation in the host. However, the ethical and religious implications of fetal tissues are still crucial issues [103]. The dangers of stem cell therapies, such as graft overgrowth and non-neuronal cells within grafts, should be considered.

A study on the brains of murine models of HD by Ebert et al. observed that mouse-derived NSCs act as GDNF delivery vehicles, playing a beneficial role in reducing neuronal death and the resultant motor impairment [104]. To address the role of environmental enrichment in stem cell therapy for HD, engineered NPCs that overexpress GDNF were transplanted into HD rodents. Although unmodified NSCs showed no neuroprotective effects, NPCs expressing GDNF afforded neuron protection and functional recovery. 

At present, MSCs offer a promising source of cells for treating HD because of their ability to decrease immune cell dysfunction, enhance compensatory neurogenesis, reduce apoptosis, activate mitochondrial function, and promote cell survival [103].

In 2010, Dey et al. reported that MSCs genetically engineered to overexpress BDNF or NGF decreased behavioral deficits and neuronal loss in the striatum in the YAC 128 mouse model of HD [105]. Thus, transplantation of MSCs overexpressing BDNF may provide a conducive environment within the striatum to slow neurodegenerative processes [9]. Snyder et al. discovered that implanting human-derived MSCs into the dentate gyrus of the hippocampus of mice models of HD could enhance the proliferation and neural differentiation of endogenous NSCs [106]. Moreover, Lin et al. showed that human-derived MSCs offered neuroprotection and neurorestoration through neural differentiation, neurotrophic support capability, and antiapoptotic effects. The result was a significant reduction in motor dysfunction in a mice HD model [107]. Other studies also demonstrated that dental pulp stem cells might be a potential therapeutic source with less post-transplant immune rejection for HD treatment [108]. Similar functional benefits of NPC striatal injections into HD rodents were also demonstrated [8]. These include NPC incorporation into and migration to secondary sites associated with HD disease, resulting in functional improvements.

There are only two reported studies investigating iPSC-derived NSCs for cell replacement therapy for HD. In the first study, Jeon et al. transplanted hiPSC-derived NSCs generated from an HD patient with 72 CAG repeats into an HD mice model and reported an improved functional outcome and no aggregates of human mutant huntingtin (mHTT) in transplanted cells [109]. In subsequent research, mouse iPSC-derived NSCs were transplanted into the lateral ventricle of normal mouse brains and indicated the aggregation of mHTT after 33 weeks. Thus, it seems that the autologous transplantation of HD patient-derived cells carrying the HD mutation causes the HD phenotype and cell death persistence. In the second study, An et al. modified an HD-patient-derived iPSC mutation and subsequently generated human neural stem cells (hNSCs) for transplantation into an HD mice model. The transplanted cells not only survived but successfully differentiated into motor neurons [110].

To date, stem cell therapy is still far away from the clinical setting for the treatment of HD since almost all the studies have been conducted in animal models. More in-depth, comprehensive preclinical studies will be needed to confirm its therapeutic potential.

### 3.4. Stem Cell Therapy in Amyotrophic Lateral Sclerosis

ALS (also known as Lou Gehrig’s disease) is a progressive, incurable neurodegenerative disease characterized by motor neurons’ deaths in the spinal cord’s ventral horn and the motor cortex. With the disease progression, symptoms such as motor weakness, twitching, stiffness, and loss of voluntary movement control become apparent [9,52]. The average progression of ALS from onset to death is 20 to 48 months. For most of the last two decades, the mutation of Cu–Zn superoxide dismutase 1 (SOD1) was the only genetic aberration associated with familial ALS onset. Studies have unveiled additional abnormalities that may be related to the onset of sporadic and non-SOD1 familial ALS [56]. These include the impairment of neuronal cytoskeletal function, protein instability, aggregation and degradation of RNA/DNA-binding proteins, and the detrimental roles of non-neuronal cells (such as astrocytes) that can be toxic and degenerative to motor neurons [111]. Several factors cause difficulty in finding effective therapies for ALS. Most of the cases are sporadic, with a combination of genetic mutations and/or presumed environmental variables. Thus, the diversity of potential causes of the disease means that any therapy would only be effective on a specific subset of patients. Unfortunately, despite the advent of modern medicinal chemistry, there is only one FDA-approved treatment, Riluzole, which provides modest therapeutic effects [112].

Stem cell therapies have generated widespread interest as a potential therapeutic approach. However, no conclusive results have yet been reported, either from preclinical or clinical studies. The unknown pathogenesis and the lack of proper knowledge of the disease’s spreading mechanism in the human body are among the most critical barriers to overcome regarding choosing the ideal cell type and optimal anatomical site for implantation.

Theoretically, the underlying strategies for treating ALS using stem cell therapies are similar to those previously described for other neurodegenerative diseases: (1) replace the damaged/dead motor neurons, (2) regulate inflammation, and (3) promote the expression of neurotrophic factors. The treatment’s end goal is equally similar to those previously described: provide both an integrated neural component and the necessary environmental enrichment to prevent existing motor neurons from degenerating [8]. The first FDA-approved clinical trial to use fetal-spinal-cord-derived NPCs in ALS patients was initiated in 2010 at Emory University. The trial’s scope was to assess the safety of implanting NSCs into the spinal cord of 18 affected patients [113]. Then, Martinez et al. assessed stem cell transplantation’s safety in the frontal motor cortex of 67 patients with ALS [114]. Numerous clinical trials on stem cell therapy for ALS evaluated the safety and feasibility of intraspinal, intrathecal, and intracerebral MSC transplants [115]. For example, surgical implantation of MSCs into the dorsal spinal cord also showed no immediate or long-term complications during a follow-up period of 9 years [116]. Indeed, these trials provided significant insight into the safety and feasibility of autologous MSC-based therapies in ALS patients [117]. Besides safety, the efficacy of the treatment needed to be evaluated. A study on 11 ALS patients in Spain demonstrated increased motor neuron numbers and reductions in the presence of ubiquitin deposits in motor neurons following MSC transplantation [118]. Current clinical trials are also focused on the exogenous transplantation of NSCs due to valid data indicating the slow progression of ALS upon the injection of fetal NSCs into the spinal cord of patients [119]. The crucial neuroprotective effect of growth factors on the remaining motor neurons and the stem cells’ ability to provide environmental enrichment through growth factor expression is currently being evaluated. For example, Brainstorm Therapeutics is developing an approach to inject MSCs into the fluid surrounding the brain and spinal cord (via intrathecal administration) of ALS patients. The study’s scope is to assess the beneficial effects of the neurotrophic pro-angiogenic, anti-inflammatory, and immunomodulatory factors secreted by MSCs [120]. 

Altogether, although stem cell therapy in ALS treatment is still in its infancy, researchers worldwide hope treatments like this will stop the disease’s progression and improve the efficacy of current drug therapies [121].

### 3.5. Stem Cell Therapy in Frontotemporal Dementia

FTD, an insidious neurodegenerative clinical syndrome, is the second-most-common leading cause of neurodegenerative dementia and is usually found in people younger than 65 [122,123,124]. FTD is characterized by progressive deficits in personality, cognition, behavior, and language functions caused by the selective death of cerebral cortex neurons and neurodegeneration of the frontotemporal cortex [125,126]. FTD clinically overlaps with some motor disorders, including progressive supranuclear palsy (PSP), corticobasal syndrome (CBS), parkinsonian disorders (FTD-PD), and motor neuron disease (FTD-MND or FTD-ALS) [127]. FTD is a genetically and pathologically complex disorder occurring in both familial and sporadic forms [125]. Statistically, familial forms of FTD represent about 20–30% of FTD and up to 15–20% of these patients carry mutations in the *MAPT* gene encoding the microtubule-associated protein tau [128,129,130,131]. The growing prevalence of FTD with the lack of treatments and the burden on society make FTD a public health priority. Therefore, it is crucial to unravel the pathological mechanisms of FTD to identify intervening biomarkers to establish more useful diagnostic guidelines and discover novel therapeutic targets. The genetic origin of a significant proportion of the familial and sporadic forms of FTD is still unknown. However, genetic mutations in tau (*MAPT*), progranulin (*PGRN*), and *C9ORF72* are among the most common causes of FTD known up to now [132]. These genes have recently been targeted for studying pathological mechanisms and discovering new pharmacological interventions for FTD. However, despite useful research findings over the past two decades, the FTD mechanisms are still poorly understood. This might be due to the lack of appropriate disease models that accurately recapitulate FTD’s complex pathologies. Moreover, there are still challenging and misguided cases in the clinical diagnosis of sporadic FTD that rely on clinical diagnostic criteria. The generation of appropriate models for investigating FTD’s molecular mechanisms has been challenging, as cell lines and animal models do not recapitulate the complex pattern mutations seen in the adult human CNS. Moreover, many studies on tau overexpression models can lead to extreme phenotypes that cannot truly reflect endogenous tau expression in FTD [127]. Interestingly, the ability to reprogram somatic cells into iPSCs may provide an attractive model for studying the pathological mechanism of FTD [133]. iPSCs have become a useful tool to recapitulate FTD patients’ disease phenotypes to elucidate the pathogenic mechanisms and accelerate drug discovery [123]. Several studies have recently reported the generation and characterization of familial FTD-patient-derived iPSCs [134]. For example, as reported by Lee et al., FTD-diagnosed patients donated peripheral blood mononuclear cells (PBMCs). Then iPSCs were developed using integration-free CytoTune-iPSC Sendai reprogramming factors, including Sendai virus particles of Oct, Sox2, Klf4, and c-Myc (Yamanaka factors) [135]. In two distinct studies, Rasmussen et al. also successfully established iPSCs from skin fibroblasts of patients diagnosed with FTD carrying R406W and P301L mutations in *MAPT* to study hereditary FTD and tau pathologies in vitro [136,137]. Nimsanor et al. also generated iPSCs from FTD patients carrying an S305I mutation in *MAPT* [138]. Almeida et al. generated multiple iPSCs from a control subject, a patient with sporadic FTD, and an FTD patient with a novel heterozygous *GRN* mutation [139]. They successfully identified cell-autonomous, reversible defects in patient neurons with Grn-deficiency and provided an applicable model to study *GRN*-dependent pathogenic mechanisms and develop potential therapies. Ehrlich et al. also derived iPSCs from patients with FTD-associated *MAPT* mutations and differentiated them into mature neurons to provide an in vitro model for identifying distinct neurodegenerative changes in frontotemporal dementia with parkinsonism-17 (FTDP-17) [140]. In another study, Kim et al. generated patient-specific iPSC lines from two sporadic FTD patients using their PBMCs and investigated the expression of pathological markers, including FTD-tau, transactive response DNA-binding protein 43 (TDP-43), active caspase-3, and fused in sarcoma (FUS) [141]. Based on their immunocytochemical and immunoblot analyses, the active caspase-3 expression was significantly elevated compared with controls. This neurodegenerative feature of FTD can be used as a potential biomarker to identify pathological mechanisms and therapeutical screening. Raitano et al. also evaluated specific neuronal cells and cortical neurons developed from iPSCs derived from FTD patients to generate a realistic FTD model characterized by selective frontotemporal cortex neurodegeneration [142]. Altogether, although the use of stem cell technology for FTD modeling is still in its infancy, researchers aim to utilize iPSCs as a beneficial tool to facilitate understanding of disease mechanisms and develop treatment strategies.

## 4. Challenges and Future Directions

Despite the remarkable advances in stem cell research for neurodegenerative disorders, several critical issues must be addressed. A major hindrance in stem cell therapies’ progression is learning how stem cells work in the body and how they integrate with the targeted tissue/organ. Furthermore, generating specialized cell typologies to overcome tissue- and environmental-specific hurdles are critical requirements. The safe and cost-effective generation of these cells in adequate quantities is also another factor to be considered. For example, ESCs and iPSCs can be grown indefinitely in the lab, but the procedures are very complex and demanding, limiting these cells’ overall availability.

Furthermore, there is the problem of reducing the risk of post-implant rejection, which adds the burden of needing a close compatible donor for the cells’ recipient. Identifying the proper conditions to culture these cells, the most suitable route of administration, delivery, and the target site is also crucial to maximizing the benefit of the treatment and improving the outcome. Unfortunately, most of the data available to researchers were derived from animal studies; therefore, it is unclear whether these human stem cells will afford similar results when administered to a heterogeneous patient population [9]. Unfortunately, animal models are designed to maximize reproducibility at the cost of neglecting the intrinsic variability and diversities between human patients. Directly extrapolating the results from these in vivo studies into human patients is not feasible at this stage. Therefore, animals’ structural and functional improvements in these experiments need to be validated in clinical trials [143].

On the other hand, translating stem cell therapy to clinical trials is practically impossible without a suitable stem cell source for therapeutic applications. The cost, time, and labor-intensive nature of stem cell therapy limit its use, especially in developing countries. Additionally, safety considerations, such as the potential for malignant transformation and side effects, such as epilepsy, immune allergic reactions, and injection site injuries, remain significant concerns [144].

The outcome of stem cell therapy can be improved by combining adjunct treatments [48]. For example, stem cell therapy coupled with erythropoietin administration demonstrated synergistic neurogenesis effects in a rat model. Nanoparticle-based delivery systems are being studied to address the shortcomings of stem cell migration and integration into functional networks [145,146,147,148]. Nanoparticles are useful in drug and cell delivery systems due to their ability to cross the BBB and reach the targeted brain regions without damaging the surrounding areas. Another alternative to facilitate the delivery and the retention of the stem cells in the transplantation site involves using encapsulation with hydrogels made of hydrophilic polymers, thus providing mechanical support in the delivery processes and increased proliferation and differentiation [149,150,151]. Recent research studies have also utilized gene therapy and neural growth factors to prolong transplanted stem cells’ retention in AD and PD [152]

Despite all the limitations and challenges, stem cell therapy is still a promising approach for treating neurodegenerative diseases in the future [18]. The use of stem cells in neurodegenerative diseases to replace lost neurons and integrate them into existing neural circuitry still seems an unrealistic and long-distant goal [143]. However, using stem cells to deliver therapeutic factors and/or preventing the disease progression appears to be a more realistic and short-term achievable goal. Additional data from ongoing and future clinical trials will provide important insights into the proper delivery approach, immunosuppression, graft survival, and efficacy [113]. With the establishment of best practice guidelines for stem cell therapies, it will be possible to develop novel cellular sources and develop more effective combinatorial approaches to treating neurodegenerative diseases. 

## 5. Conclusions

Neurodegenerative diseases have devastating sequelae with conventional pharmacological therapies. To date, progress in the research area of stem cell therapies offers excellent promising methods for the treatment of these disorders. Although much work remains to be done, the growing focus on preclinical studies and the recent translation of some of these therapies to clinical trials has set the stage to continue progress. Using stem cells appears likely to become a key feature of future clinical strategies for treating neurodegenerative disease by replacing dysfunctional neurons and affording neuroprotective and neurorestorative functions. Furthermore, stem cells’ recent technological developments involving nanoparticles and hydrogels have made drug delivery and regeneration treatments more efficient. Thus, neural replacement and regenerative therapies are soon expected to be successfully translated into the clinical setting.

## Figures and Tables

**Table 1:** Comparison between the various types of stem cells. This side-by-side comparison includes their origin and the inherent clinical advantages and disadvantages of using these cells.

Stem Cell Type	Origin	Advantages	Disadvantages
ESCs (pluripotent)	Embryo (blastocyst)	✓ Unlimited proliferation	✓ Ethical problems ✓ Risk of immune rejection ✓ Unpredictable differentiation ✓ High risk of tumor formation
IPSCs (pluripotent)	Reprogrammed adult cells: fibroblasts, hepatocytes, circulating T cells, and keratinocytes	✓ No ethical problems ✓ Low risk of immune rejection ✓ High accessibility	✓ High risk of tumor formation ✓ Risk of susceptibility to the original pathology of the patient ✓ Genetic and epigenetic abnormalities
MSCs (multipotent)	Adult tissues (bone marrow, skin, blood, umbilical cord, etc.)	✓ No ethical problems ✓ High accessibility ✓ Easy isolation methods ✓ Autologous cells generation ✓ Self-renewal capacity ✓ Low risk of immune rejection	✓ Risk of tumor formation
NSCs (Multipotent)	Embryo, human fetal brain and brain tissue of adults (SVZ and SGZ of hippocampus)	✓ Low risk of tumor formation	✓ Ethical problems ✓ Risk of immune rejection ✓ Limited differentiation ✓ Low self-renewal capacity ✓ Limited proliferation and expansion ✓ Limited availability ✓ Difficult isolating methods

SGZ: subgranular zone, SVZ: subventricular zone.

## Data Availability

Data sharing not applicable. No new data were created or analyzed in this study. Data sharing is not applicable to this article.

## Abbreviations

AD	Alzheimer’s disease
ALS	amyotrophic lateral sclerosis
BDNF	brain-derived neurotrophic factor
CNS	central nervous system
DA	dopamine
ESCs	embryonic stem cells
FTD	frontotemporal dementia
GDNF	glial-cell-line-derived neurotrophic factor
HD	Huntington’s disease
IPSCs	induced pluripotent stem cells
MSCs	mesenchymal stem cells
NFTs	neurofibrillary tangles
NGF	nerve growth factor
NPCs	neural progenitor cells
NSCs	neural stem cells
PD	Parkinson’s disease
ROS	reactive oxygen species
SGZ	subgranular zone
SVZ	subventricular zone
